# Transition Dependency: A Gene-Gene Interaction Measure for Times Series Microarray Data

**DOI:** 10.1155/2009/535869

**Published:** 2009-01-05

**Authors:** Xin Gao, Daniel Q Pu, Peter X-K Song

**Affiliations:** 1 Department of Mathematics and Statistics, York University, 4700 Keele Street, Toronto, ON, Canada, M3J 1P3; 2 Department of Biostatistics, University of Michigan School of Public Health, Ann Arbor, MI 48109-2029, USA

## Abstract

Gene-Gene dependency plays a very important role in system biology as it pertains to the crucial understanding of different biological mechanisms. Time-course microarray data provides a new platform useful to reveal the dynamic mechanism of gene-gene dependencies. Existing interaction measures are mostly based on association measures, such as Pearson or Spearman correlations. However, it is well known that such interaction measures can only capture linear or monotonic dependency relationships but not for nonlinear combinatorial dependency relationships. With the invocation of hidden Markov models, we propose a new measure of pairwise dependency based on transition probabilities. The new dynamic interaction measure checks whether or not the joint transition kernel of the bivariate state variables is the product of two marginal transition kernels. This new measure enables us not only to evaluate the strength, but also to infer the details of gene dependencies. It reveals nonlinear combinatorial dependency structure in two aspects: between two genes and across adjacent time points. We conduct a bootstrap-based  test for presence/absence of the dependency between every pair of genes. Simulation studies and real biological data analysis demonstrate the application of the proposed method. The software package is available under request.

## 1. Introduction

Biological processes in the cell such as biochemical interactions and regulatory activities involve complicated dependency relationships among genes. It is one of the most fundamental aims in biology to build up appropriate models for inferring such dependency relationships. Time series microarray data consist of trajectories of gene expression profiles at multiple time points, which provide an innovative platform for biologists to investigate the dynamic nature of gene dependencies. Such gene-gene dependencies are attributed to some physical interactions among encoded proteins or between an encoded protein and genes, or through coregulation of some common transcription factors. Although from the microarray data, we cannot directly learn about how these physical interactions work, we can still make inference whether or not there is a dependency relationship between two genes' transcriptional changes via some mathematical models. The notion of gene-gene interaction in this article refers to such dependency relationship in the expression levels.

Many methods have been proposed to detect gene-gene interactions using microarray data [1–3]. A traditional approach is to cluster genes using pairwise Pearson or Spearman correlations as a distance measure [4–6]. Pearson correlation captures linear dependencies and depends on normality assumption. Spearman correlation measures the concordance in the ranks of data and is invariant to any monotonic transformations on the data. As it does not rely on any normality or linearity assumptions, it is often used as a robust statistic to identify the coexpression patterns in genes. When applied on a pair of time series data, calculating both Pearson and Spearman correlations implicitly assumes that all the paired measurements across different time points are independent replications. This calculation is too simplistic to adequately describe the complex relationship between two time series, in which the dependency may be beyond a linear or monotone pattern. In the literature, there are several extensions of Pearson correlation in the context of time series data. For example, Dubin and Müller [7] introduced the notion of dynamic correlation (DC) across two time series, which, however, is not sensitive to autoregressive dependency. Another commonly used correlation measure in time series is cross-correlation function (CCF) proposed in [8], which calculates a linear correlation across lagged time points. Nevertheless, neither DC nor CCF is deemed to measure nonlinear dependencies.

In this article, we invoke hidden Markov models (HMMs) that give rise to a gene-gene dependency measure. The HMMs framework allows us to make a few new developments that overcome some of the key difficulties in the existing methodologies discussed above. We propose a new dependency measure based on transition probabilities across two Markovian processes, which allows us to study nonlinear relationships among genes. An intuition behind the proposed approach is that we intend to track time-varying behaviors of interactions among genes. This dynamic relationship seems naturally reflected by the transitional mechanism described in the HMMs. Thus, the dependency between two genes can be characterized via the difference between their joint transition matrix and the product of the two corresponding marginal transition matrices. In spirit, this idea is very similar to the concept of mutual information (MI) [9], which measures the difference between the sum of marginal entropies and the bivariate joint entropies. When the two random variables are independent, the MI takes zero value. Both approaches are based directly on probability arguments and both can detect nonlinear relationships among interacting genes. Unfortunately, the MI is only defined for two random variables and cannot be readily applied to time series data. In contrast, the proposed transition dependency is developed specifically to evaluate nonlinear dependencies between two time series. As shown in Section 2, this dependency measure is rich in detail describing how a pair of genes influence each other over time. We will use this dependency measure to perform a screening analysis that selects significant pairwise dependencies among all the gene pairs at a reasonable false discovery rate. The related statistical significance is given by a bootstrap-based -test.

## 2. Method

### 2.1. Definition of Transition Dependency Measure

We now introduce a new dependency measure across two Markovian processes. Consider a bivariate HMMs with discrete hidden states. Let the collection of bivariate hidden states be  where  for a pair of genes. Given the hidden state  or  the conditional distribution of  is denoted as  or  respectively. Here, depending on the observation process , the hidden state may have different interpretations. For a one-sample experiment,  could stand for a normalized measurement of gene expression level or hybridization intensity, and the corresponding hidden states may be labelled as "upregulated" (UR = 1) and "downregulated" (DR = 0), respectively. In the context of two-sample comparative experiment,  could stand for a measurement of difference in expression values across two experiment conditions for gene  at time  Then, the hidden states  can be regarded as "differentially expressed" (DE = 1) and "not differentially expressed" (NDE = 0) as in [10]. Many methods are available to estimate the conditional distributions  and  including nonparametric empirical Bayes method in [11], parametric empirical Bayes method in [10], and EM method for finite mixture models [12].

Suppose that the bivariate hidden states follow a stationary Markovian process, and the joint transition matrix is denoted as  with  In this HMMs framework, we define a measure of dependency across two univariate processes as follows:(1)

with  and  denoting the two marginal transition matrices and  denoting the Kronecker product of two matrices. This transition dependency matrix  measures the deviation of the actual joint transition matrix from the expected joint transition matrix under the independence assumption. It has been proved by Sandland [13] that if the two processes are independent, then all the entries of matrix  should be equal to zero. In other words, when two processes are dependent, this cross-dependency matrix  would fully characterize the strength of their dependency. The continuous analog of this dependency measure between two point processes has been proposed in [14].

To interpret the transition dependency matrix , here we give two examples.

*Example 1*. Each entry of the dependency matrix  corresponds to the dependency in different direction and has its own biological interpretation. For instance, if the hidden states of DE  and NDE  satisfy  then gene 2 has an *induction effect* on gene 1. This means that the DE state of gene 2 enhances the probability of gene 1 switching from NDE state to DE state. The contrary is *inhibition effect*, where the hidden states satisfy  This implies that the DE state of gene 2 reduces the probability of gene 1 changing from NDE state to DE state.

*Example 2*. This example shows that the proposed transition dependency is able to capture some nonlinear dependency relationships but the traditional linear correlation fails. Suppose the hidden states represent DR and UR categories, respectively, with the joint transition matrix between genes 1 and 2 given by(2)

**Figure 1:**
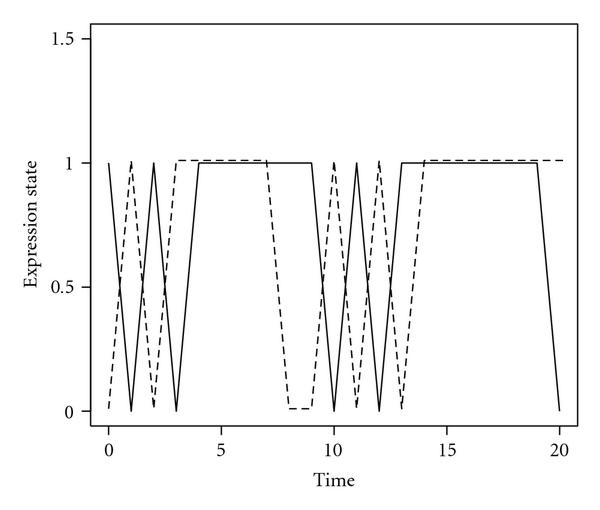
**Simulated expression status of RNA upstream and downstream configurations**.

It is easy to show that the stationary distribution of the resulting Markov process is  which leads to an expected zero value of the dynamic correlation between the two marginal processes  and  Therefore, the dynamic correlation will not be able to detect any dependency between these two processes. As a matter of fact, the two-time series mutually influence each other in order to reach an equilibrium state. That is, if they are both in DR or both in UR, they tend to remain at the same state; if not, say, one of them being in DR and the other in UR, then they tend to induce the DR gene and suppress the UR gene. This type of biological regulation for achieving and maintaining the equilibrium state is often observed between RNA upstream and downstream configurations [15]. Figure 1 displays two simulated trajectories according to the given joint transition matrix (2).

The sample dynamic correlation for the simulated data depicted in Figure 1 is , indicating no linear correlation between the two processes. In contrast, the Kronecker product of two marginal transition probabilities is given by(3)

It is evident that there is a large discrepancy between the joint transition matrix (2) and the product of the marginal transitions (3). The resulting nonzero matrix  provides the evidence for a strong dependency between the two genes. The failure of the traditional correlation measure to detect the dependency here is due to the fact that it essentially relies on the concordant and discordant changes between two trajectories which are clearly absent in this type of nonlinear dependency relationship.

### 2.2. Testing for Pairwise Dependency

Consider a statistical test for the absence or the presence of interaction between two genes. The null hypothesis is , where  is a  matrix of all elements equal to zero. Pearson-type  test is popular to test for independence, and our proposed test will follow on this line. As a first step to construct a test statistic, we need to obtain the maximum likelihood estimate (MLE) for the transition matrix. Assume there are  replications of the observed data  with  and  where the first subscript indexes for the observations of gene 1 and gene 2, and the second subscript indexes for the time point. Let  denote the set of four possible configurations of the joint hidden states for  Denote the distribution of the bivariate state vector at the initial time by  with . Then, the augmented likelihood of the "complete" data with a given transition matrix takes the following form:(4)

The maximum likelihood estimates of the unknown parameters  can be obtained as(5)

As the hidden state vectors  are unobserved, the EM algorithm is invoked to carry out the maximum likelihood estimation, which iterates the following two steps till convergence.

E Step: given , we calculate two conditional expectations that are the expected numbers of transitions:  and  This is achieved by using the forward-backward algorithm especially designed for the HMMs model [16]. 

M Step: given these expected numbers of transitions between the states, we update the transition matrix by the following MLE: (6)

As usual, multiple starting points can be used to achieve the global maximum instead of local stationary points. To test for the null hypothesis , we can tabulate relevant data in a form of contingence table, where cell count  denotes the total number of transitions between states  and  Let  be the number of marginal transitions from  to  for gene 1, and let  be the number of marginal transitions from  to  for gene 2, with  or  Under the , the expected frequency of transitions is  where  denotes the th element of vector  or  Thus a chi-squared-type test statistic [17] can be formed as 

Even when the s are available, because of the autocorrelations between the transitions across time points, the limiting distribution of  is not a chi-squared distribution of 9 degrees of freedom. Furthermore, all the counts  are not observed, we have to estimate them. Upon the convergence of the EM algorithm, we may obtain the estimated counts of transitions between each pair of states:  The resulting statistic is denoted by , with  in place of  in the  statistic. Thus the estimation procedure brings extra random variation into the statistic .

To assess the significance of  statistic, we invoke the bootstrap method to generate its empirical null distribution. We randomly resample the bivariate hidden Markovian process under the null hypothesis (cross-independence) as follows. From the EM algorithm, we estimate the marginal transition matrices under the null hypothesis. For each run of bootstrap sampling, using  and the estimated marginal transition matrices, we randomly generate  bivariate Markovian processes where the two processes of hidden states are cross-independent. Based on the sample path of the  we then randomly generate the measurement process  according to the conditional distributions. Subsequently, we discard , treat the generated  as the bootstrap data, and invoke the EM algorithm. Utilizing the output of the EM estimates based on the bootstrap data, we can calculate a value of  statistic, which can be viewed as a random draw from the null distribution of the statistic. By generating a large number of bootstrap replicates, we can obtain the empirical distribution of the null statistic which provides an accurate approximation to the null distribution of  statistic.

### 2.3. Pairwise Analysis

In microarray data, the expression trajectories of  genes can be modeled as an -variate times series data,  where  indexes for the sample replicate,  indexes for the th variate (gene), and  indexes for the time point. In practice, two kinds of pairwise analyses may be considered: (1) given a specific gene of interest, and the task is to infer all the genes that interact with this gene; (2) test all  pairs exhaustively, and select the most significant pairwise dependencies for a further analysis.

In both scenarios, a list of potentially promising interactions are determined while the false discovery rate (FDR) is under control. False discovery rate (FDR) is an error measure used in the context of multiple hypotheses testing. Given a family of  simultaneously tested null hypotheses of which  are true. Let  denote the number of rejected hypotheses, and let  denote the number of true hypotheses erroneously rejected. Let  denote  when  and  otherwise. Then the FDR is defined as , the expected rate of false discovery. As shown in [18], the FDR of a multiple comparison procedure is always smaller than or equal to the familywise error rate (FWER). To control the FDR, we proceed as follows. For each pair  we construct the  test statistic, and also generate bootstrap-based null statistics  To deal with the issue that test statistics are correlated, we follow Reiner et al. [19] to form the null distribution by collapsing all the null statistics together. Thus the -value of each pairwise test can be obtained by referring to the empirical null distribution. Given the ordered -values,  the multiplicity adjusted -value employed by the Benjamini-Hochberg (BH) procedure [18] is  where  denotes the total number of tests under screening. Pairs with adjusted -values less than a prespecified FDR are declared to be significant and selected for a further consideration. Although this screening procedure potentially contains some false positives, it is computationally efficient and provides a promising pool of candidate relationships for a future analysis.

## 3. Results on Simulated Data

A simulation study was conducted to investigate the empirical performance of the proposed bootstrap-based test for pairwise gene dependency. One thousand pairs of genes were simulated under different transition probabilities. Under the null hypothesis  of independence, the underlying transition matrix takes the form(7)

where the parameters satisfy , and , , , , with  denoting a uniform distribution. To specify the alternative hypothesis, we considered a deviation drift  which deviates the null transition matrix  according to the following two patterns: Pattern I takes the form(8)

and Pattern II takes the form(9)

In our simulation study, a few scenarios were given via the combinations of different parameter values, including the deviation parameter  and , the number of replicates , and , and the number of time points  and . For each pair of genes,  or  bootstrap samples were generated to form the null statistics, and they were then collapsed together to form the empirical null distribution [19]. The conditional distributions  and  were chosen to be  and , respectively. To test the null hypothesis , our HMMs approach was compared with two correlation-measure-based methods, namely, the sample dynamic correlation (DC) method and the classical cross-correlation function (CCF) method in the theory of multivariate time series analysis. Both DC and CCF methods used their respective empirical distribution from the bootstrap samples to obtain the corresponding -values under the null hypothesis .

Tables 1 and 2 provide the empirical type I error rates and the power of these three competing methods under the two different dependency patterns over 1000 simulations. Type I error rates given by all the three methods (with ) were reasonably controlled at the  level. Comparing the power across these three methods, we can see that the bootstrap-based  test (BS-) clearly outperformed the other two methods. Under the alternative  of Pattern I, the BS- method maintained fairly satisfactory power, which was always better than the two correlation-measure-based methods. Under the alternative  of Pattern II, it is interesting to see that the two correlation-measure-based methods had no power of detecting the dependency here. Their power values were constantly around , regardless of the size of dependency (i.e., deviation ). In contrast, the power of the BS- method responded well to the increase in deviation .

**Table 1:** Empirical type I error rates and power of the proposed bootstrap-based (BS)  test versus the dynamic correlation (DC) and cross-correlation function (CCF) to detect pairwise dependency under the dependency pattern I.

				
Replicates	Time points			DC	CCF		DC	CCF
2	7	0.00	0.084	0.057	0.038	0.070	0.054	0.039
		0.05	0.135	0.092	0.038	0.120	0.077	0.045
		0.10	0.249	0.198	0.061	0.260	0.183	0.073
		0.15	0.472	0.388	0.098	0.448	0.369	0.092

2	10	0.00	0.054	0.053	0.049	0.044	0.045	0.033
		0.05	0.131	0.101	0.052	0.118	0.113	0.045
		0.10	0.281	0.256	0.081	0.298	0.286	0.100
		0.15	0.583	0.561	0.150	0.577	0.561	0.157

3	7	0.00	0.071	0.055	0.043	0.058	0.053	0.051
		0.05	0.118	0.120	0.056	0.127	0.109	0.058
		0.10	0.302	0.284	0.109	0.313	0.288	0.110
		0.15	0.594	0.586	0.168	0.564	0.567	0.144

3	10	0.00	0.049	0.058	0.042	0.060	0.052	0.059
		0.05	0.163	0.131	0.072	0.133	0.127	0.061
		0.10	0.401	0.388	0.123	0.396	0.384	0.135
		0.15	0.766	0.735	0.253	0.754	0.732	0.256

5	7	0.00	0.056	0.051	0.036	0.060	0.050	0.037
		0.05	0.172	0.141	0.073	0.165	0.133	0.066
		0.10	0.488	0.478	0.155	0.468	0.452	0.153
		0.15	0.822	0.823	0.298	0.843	0.841	0.294

5	10	0.00	0.042	0.070	0.051	0.054	0.038	0.052
		0.05	0.231	0.196	0.087	0.218	0.198	0.083
		0.10	0.624	0.648	0.227	0.638	0.647	0.260
		0.15	0.946	0.938	0.456	0.949	0.953	0.463

The power refers to the probability of detecting the interaction when the interaction really exists. The symbol  denotes the number of bootstrap samples generated for each gene. The symbol  denotes the deviation parameter.

**Table 2:** Empirical type I error rates and power of the proposed bootstrap-based (BS)  test versus the dynamic correlation (DC) and cross-correlation function (CCF) to detect pairwise dependency under the dependency Pattern II. The power refers to the probability of detecting the interaction when the interaction really exists.

				
Replicates	Time points			DC	CCF		DC	CCF
2	7	0.00	0.084	0.057	0.038	0.076	0.038	0.032
		0.05	0.077	0.048	0.036	0.066	0.051	0.042
		0.10	0.078	0.053	0.045	0.095	0.059	0.041
		0.15	0.125	0.046	0.038	0.122	0.044	0.038

2	10	0.00	0.059	0.053	0.047	0.058	0.033	0.039
		0.05	0.087	0.050	0.040	0.063	0.039	0.043
		0.10	0.086	0.047	0.032	0.102	0.043	0.040
		0.15	0.152	0.049	0.039	0.157	0.048	0.037

3	7	0.00	0.059	0.052	0.028	0.074	0.045	0.040
		0.05	0.085	0.048	0.045	0.070	0.049	0.042
		0.10	0.106	0.057	0.052	0.099	0.052	0.040
		0.15	0.137	0.037	0.031	0.137	0.053	0.041

3	10	0.00	0.049	0.054	0.041	0.045	0.041	0.045
		0.05	0.081	0.050	0.042	0.051	0.047	0.043
		0.10	0.116	0.048	0.037	0.126	0.040	0.032
		0.15	0.222	0.045	0.036	0.217	0.043	0.051

5	7	0.00	0.065	0.049	0.035	0.059	0.056	0.050
		0.05	0.073	0.055	0.044	0.071	0.057	0.051
		0.10	0.131	0.058	0.044	0.114	0.050	0.043
		0.15	0.203	0.053	0.052	0.239	0.049	0.039

5	10	0.00	0.042	0.049	0.048	0.052	0.049	0.047
		0.05	0.094	0.058	0.058	0.064	0.045	0.040
		0.10	0.186	0.044	0.054	0.181	0.041	0.049
		0.15	0.475	0.058	0.042	0.516	0.060	0.054

The symbol  denotes the number of bootstrap samples generated for each gene. The symbol  denotes the deviation parameter.

Why did the two correlation-measure-based methods perform well under the dependency Pattern I, but very poorly under Pattern II? This is because the correlation essentially measures the discordance and concordance between the joint expression states. For example, given the transition matrix under the null distribution specified by , when the deviation  increases from  to  the stationary distribution of  on the four possible pairs  and  will change from  to  under Pattern I. Apparently, such a stationary distribution allocates more probabilities on the concordance pairs , namely 0.32 and 0.37, and , namely 0.37 and 0.42. This causes high correlation easy to detect. In contrast, Pattern II behaves strikingly different. When  increases from  to , the stationary distribution of  remains almost the same, from  to . The stationary distribution takes almost equal probabilities on these four pairs. The evenly distributed concordant and discordant pairs lead to low correlations. This explains the poor power of the correlation-measure-based methods to detect dependency Pattern II.

## 4. Results on Biological Data

### 4.1. Apoptosis Data Analysis

To investigate the practical performance of the proposed method, we consider the neutrophil apoptosis microarray dataset produced by Kobayashi et al. [20]. The neutrophils are important cellular component of the innate immune system in humans. It is essential that neutrophils undergo spontaneous apoptosis as a mechanism to facilitate the stability of the immune system. To get a global view of the molecular events that regulate neutrophil survival and apoptosis, Kobayashi et al. [20] studied the global expression in human neutrophils during spontaneous apoptosis cultured with and without human GM-CSF, which is known to prolong neutrophils survival against apoptosis. Neutrophils were isolated from venous blood of three healthy individuals and were cultured in the medium with and without 100 ng/mL GM-CSF for up to 24 hours. At time points, 3 hours, 6 hours, 12 hours, 18 hours, and 24 hours, the expression level of 12 625 genes were measured using GeneChip hybridization technique. The time course data we analyzed contains 30 samples comparing treatment (+GM-CSF) versus control (GM-CSF) at the corresponding 5-time points in three biological replicates.

To use this dataset and understand the gene regulatory network, as a first step, we wish to find out how genes are interacting with each other. We selected CD44 as our gene of interest and set out to find all the genes that are interacting with CD44 during the neutrophils apoptosis. CD44 is an important gene which encodes a cell surface glycoprotein involved in cell-cell interactions, cell adhesion and migration. This protein participates in a wide variety of cellular functions including lymphocyte activation, recirculation and homing, hematopoiesis, and tumor metastasis. It is expected that CD44 interacts with a variety of genes to facilitate its various functions. Furthermore, CD44 is an important tumor marker which is released by cancerous cells and could be detected by blood tests to detect the presence of cancer. To provide a list of candidate genes which interact with CD44 can provide more insight into the biological mechanism underlying tumor progression.

To apply the proposed HMMs, we first took  to be the absolute difference between the th biological replicate's expression levels under the two experiment conditions from gene  evaluated at time . Next we need to determine the conditional distributions  and  given the NDE status and DE status. Nonparametric empirical Bayes method in [11] was employed to estimate these conditional distributions, both of which were fixed in all the subsequent hypothesis tests for computational convenience. It assumes that the underlying distribution for the statistic  is a mixture distribution containing two components:  where  represent the components corresponding to DE state  and NDE state , and  and  are the probability that an observed  is sampled from  and , respectively. Then based on  one can make posterior inference whether the specific observation is from state  or state  Unlike the classical Bayes approach, which assumes specific parametric forms of  and  the nonparametric empirical Bayes uses the data to estimate the densities of  and  First the data is randomly permuted across the two-sample experimental conditions and the null statistic is generated. By a great number of permutations, we could obtain a large random sample from  Therefore, we can estimate the densities of both  and  using nonparametric methods, such as the kernel estimation.

The gene-gene interaction was examined by testing for independence between CD44 and each of the remaining 12 624 genes. As all the test statistics are related to the expression data of CD44, all the 12 624 test statistics are not independent. To adjust for the multiplicity of the test statistics with high intercorrelations, the resampling-based FDR control method discussed above was employed. Bootstrap samples were generated to get the null distribution of the test statistics and the null statistics were collapsed to assess the *P*-values of the test statistics under the dependency structure. Then the *P*-values were adjusted for the multiplicity through the BH procedure. The significant genes were selected while maintaining the FDR control at the level of  We detected 302 significant genes having interactions with CD44 among the remaining 12 624 genes. Table 3 provides the list of the most significant 15 genes having interactions with CD44, including gene names, gene functions, and the -values. Some of these genes have existing biological evidence to directly support our findings, while some other genes have indirect evidence about the interaction between CD44 and relevant genes encoding proteins in the same protein family. Related references are included in the table as well.

**Table 3:** The list of the15 most significant candidate genes having interactions with CD44

Probe			Gene title	Literature
38336_at	0.00110891	9.505	FERM domain containing 4B (FRMD4B)	[21]
947_at	0.04692277	0.00011089	*Gene function unknown*	
39237_at	0.51548517	0.00017426	Mitogen-activated protein kinase 3 (MAPKAPK3)	[22]
40968_at	0.00367525	0.00017426	Suppressor of cytokine signaling 3 (SOCS3)	[23]
31491_s_at	0.00107723	0.00019010	Caspase 8 (CASP8)	[24]
36985_at	0.02434851	0.00020594	Isopentenyl-diphosphate delta isomerase (IDI1)	
36344_at	0.01527129	0.00022178	Coagulation factor II (thrombin) receptor-like 1 (F2RL1)	
1441_s_at	0.01954851	0.00023762	Tumor necrosis factor receptor superfamily, member 6 (FAS)	[25]
31792_at	0.00267723	0.00023762	Annexin A3 (ANXA3)	[26]
33289_f_at	0.00365941	0.00023762	Zinc finger protein 263 (ZNF263)	
953_g_at	0.01698218	0.00023762	*Gene function unknown*	
35799_at	0.02151287	0.00025347	DnaJ (Hsp40) homolog, subfamily B, member 9 (DNAJB9)	
2035_s_at	0.00327921	0.00026931	Enolase 1, (alpha) (ENO1)	[27]
31318_at	0.03653069	0.00028515	*Gene function unknown*	
296_at	0.03504159	0.00030099	*Gene function unknown*	

The results of the HMMs and the dynamic correlation methods are in agreement in most cases but differ in some cases. For the example of the third most significant gene MAPKAPK3, the estimated expected transition matrix under the null hypothesis is(10)

and the estimated joint transition matrix is(11)

The resulted  test statistics is  and the -value is . The strong dependency between the genes CD44 and MAPKAPK3 is revealed by the big discrepancy between the expected and the actual transition matrices. The joint state of the two genes has much smaller probability than expected to transit to states  and  In comparison, the estimated Pearson's correlation is only  with the insignificant -value of .

It is worthy to highlight our findings of the significant interaction between CD44 and caspase 8. Our method ranks caspase 8 as the fifth in the list with a -value of  whereas, the dynamic correlation method ranks caspase 8 as the 308th in the list with a -value of  The transition dependency matrix  was estimated to be(12)

The signs of the entries significant away from zeros are(13)

This dependency pattern is very similar to Pattern I considered in our simulation. It implies that caspase 8 and CD44 are involved in the same pathway of apoptosis and they tend to be in the same states of DE or NDE, depending on whether the pathway is initiated or not. This discovery only informs us about the existence of dependency but does not provide information about the physical mechanism. Searching through the literature, we found that this dependency is caused by the event that the CD44 encoded protein ligates with A3D8, acts as a transcription factor, and initiates the transcription of caspase 8 [24]. This discovery is of great biological implication in the sense that it unveils a new apoptosis pathway and sheds light to a potential therapeutic drug—A3D8 which ligates to CD44 and initiates caspase 8 in the pathway—to treat leukemia patients who are resistant to traditional chemotherapy agents ATRA and As2O3. Based solely on gene expression profiling without extensive wet lab work, we rediscovered that gene caspase 8's transcription level is dependent on that of CD44, with stronger statistical significance compared to the dynamic correlation method. This demonstrates the power of the proposed method of detecting biological meaningful dependencies.

**Figure 2:**
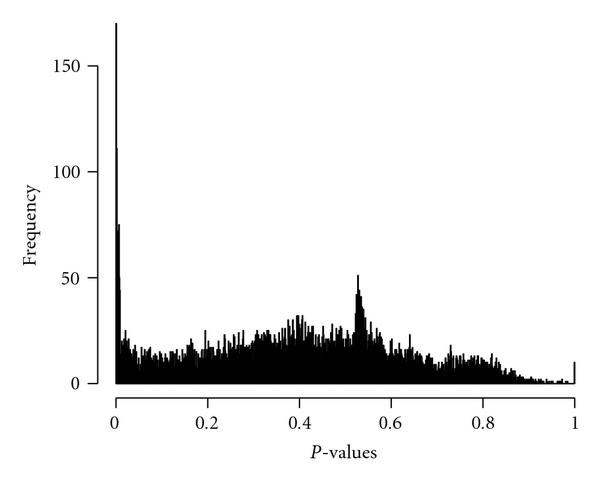
**Comparison of histograms of -values obtained from the HMMs-based transition dependency and the dynamic correlation**. HMMs method. Dynamic correlation method

To compare the overall performance of the HMMs method with the correlation method, we plotted the histograms (see Figures 2(a) and 2(b)) of the empirical -values obtained from the two methods. It is seen from Figure 2(a) that the -values from the HMMs method demonstrate a sharp spike over the range of -values less than .001. Beyond the spike, all the remaining -values follow an almost uniform distribution from .001 to 1. The proportion of -values less than .001 is 1.3%, whereas that less than 0.1 is 13.8%. The spike standing for 1.3 percent of the overall genes can be roughly viewed as the collection of genes with significant interactions with CD44, while the remaining majority of genes is independent of CD44, belonging to the null situation. In comparison, Figure 2(b) indicates that -values from the dynamic correlation method have a much lower degree of separation between the -values from the null and the alternative situations. Furthermore, there is a large bulk of -values less than .1, accounting for 47% of the overall genes, whereas the percentage of *P*-values less than .001 is only 0.3%. Thus, the dynamic correlation method identifies a large proportion of genes (almost half) being correlated with CD44 with mild statistical significance. This excessively large proportion cannot be plausibly interrelated to the proportion of the genes having real biological interactions with CD44 at molecular levels. According to the theory of sparse network held by the biologists, the HMMs method is a more reliable method to identify a small number of gene-gene interactions with biological significance.

**Figure 3:**
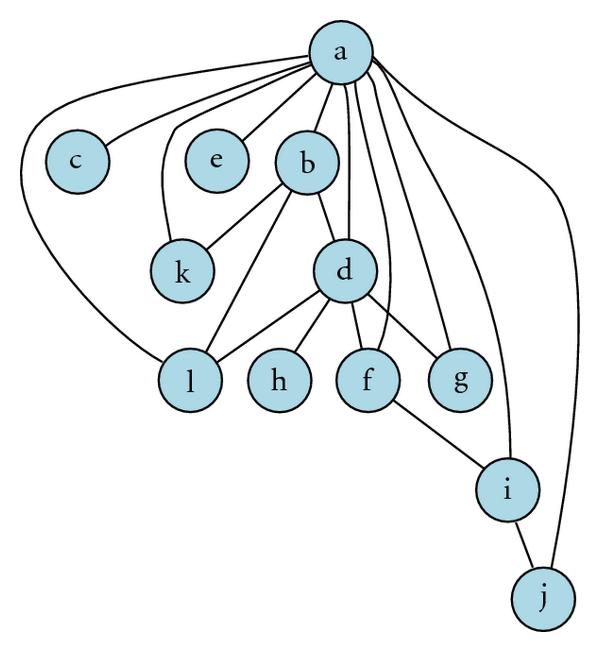
**A dependency network for CD44 and its significant relatives**. Symbol "a" stands for CD44, "b" for FRMD4B, "c" for MAPKAPK3, "d" for SOCS3, "e" for CASP8, "f" for IDI1, "g" for F2RL1, "h" for FAS, "i" for ANXA3, "j" for ZNF263, "k" for DnaJ, and "l" for ENO1.

We further investigated a full dependency map among the 15 top-ranked genes. After eliminating the four probes (947_at, 953_g_at, 31318_at, 296_at) with unknown biological functions, we relabeled the CD44 gene as gene "a", and the remaining 11 genes (FRMD4B, MAPKAPK3, SOCS3, CASP8, IDI1, F2RL1, FAS, ANXA3, ZNF263, DnaJ, ENO1) in the list as "b" to "l". The CD44 acts as a hub connecting to each of the remaining genes in the network. We obtained all the pairwise test statistics (in total 55 test statistics for 11 genes) in the network and calculated the corresponding -values via the bootstrap method. Based on the individual -value threshold of  which corresponds to the familywise type I error rate controlled at  our analysis yields a dependency network consisting of 12 nodes and 19 edges, shown in Figure 3. In the graph, an edge linking two genes demonstrates a significant dependency between them, whereas the absence of an edge means there is no significant dependency relationship between the two genes. Among the 19 edges in the network, 10 edges are supported by the existing biological evidence. According to Cheng et al. [28], there is a positive feedback loop that couples Ras/MAPK activation which involves MAPKAPK3 (node c) and CD44 (node a) alternative splicing. The presence of SOCS3 (node d) acts as a negative feedback on the activity of signaling in the JAK/STAT pathway [29], which involves ANXA3 (node i). Furthermore, the JAK/STAT pathway crosses with the Ras/MAPK pathway at multiple levels, each enhancing activation of the other [30]. Based on these biological results, we conclude that the selected network actually reflects how the three pathways—Ras/MAPKAPK3 signaling pathway, JAK/STAT signaling pathway, and caspase-dependent apoptosis pathway—interconnect with each other through the hub gene CD44.

### 4.2. T-Cell Data Analysis

**Figure 4:**
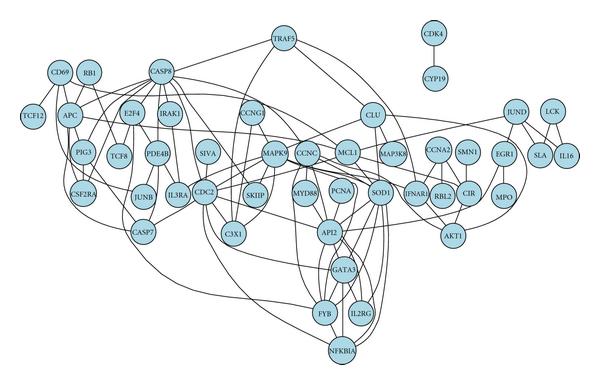
**A major dependency network of 58 genes in T-cell data analysis**.

We also analyzed the T-cell data [31] to study the genetic dependency network in the activation process of T-cells. To generate an immune response, the T-cells become activated and then proliferate, and produce cytokines involved in the regulation of B cells and macrophages, which are the most important mediators of the immune response. It is known that T-cell activation is initiated by the interaction between the T-cell receptor complex and the antigens. This stimulates a network of signaling molecules, including kinases, phosphatases, and adaptor proteins that parallel the stimulatory signals received by the nucleus to control the gene transcription events. The calcium ionophore ionomycin and the PKC activator phorbol ester PMA were used to activate signaling transduction pathways leading to T-cell activation. Microarray measurements of 58 genes which are relevant to the immune response were taken at the following times after the treatment: 0, 2, 4, 6, 8, 18, 24, 32, 48, and 72 hours. For each time point, there are 44 replicated measurements for each gene. This dataset is different from the previous apoptosis data as it is one-sample data with only one experimental condition. We used a mixture of two Gaussian distributions to model the distribution of the expression level for each gene, conditional on downregulated or upregulated state. In the pairwise analysis, we screened all possible pairs of genes exhaustively and constructed a complete dependency map for these 58 genes. For each pair,  bootstrap samples were generated to facilitate the assessment of -values. A major network is obtained, consisting of 47 genes out of the total 58, in which 89 edges have -values significant at the  level. The network is shown in Figure 4.

**Figure 5:**
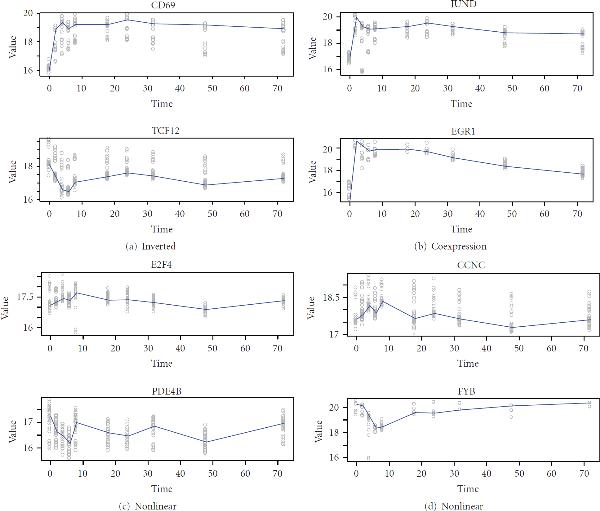
**Examples of pairwise time series identified to have interactions**. Each panel contains the time series plots, with dots representing observed replicated expression levels, and solid lines representing the average expression level across time points.

We further examined all the pairwise time series corresponding to the selected edges, some examples are shown in Figure 5. From their time series plots, we noticed that our method was capable of identifying many different patterns. The patterns include coexpression, where the two time series follow the same trend of going up or down. Another pattern is inverted, where the two time series show opposite changes over time. There exist other scenarios, where the two time series do not obey any obvious linear patterns, indicating the nonlinear combinatorial dependency relationships between the two genes. In comparison, existing methods like the linear dynamic system or the Gaussian graphical model are unable to identify such patterns.

## 5. Discussion

Detecting gene-gene interaction is one of the most important tasks in the study of system biology. The advent of time series microarray data challenges statisticians to develop a statistical machinery to extract and summarize the dependency information embedded in the data. In this paper, we characterize the dependency relationships based on the dynamics of a hidden Markov model, so that we are able to monitor the gene-gene interactions through transitional probabilities. The proposed methodology is not restricted to the microarray dataset we focus on in this article. It can be viewed as a general approach to analyze time series data with complicated dependency structure, such as brain image data and proteomics data. The method can be extended in a few directions. One limitation of the proposed method is the assumption of stationarity on the hidden process. This is more constrained by the practical limitations of small replications of microarray data rather than theoretical considerations. If the number of replications at each time point is greatly increased, we could relax the homogeneous assumption and model different transition kernels at different time points.

As requested by one of the referees, we compare our method and other existing methods in this concluding paragraph, highlighting the advantages and limitations of each method. Dynamic Bayesian networks (DBNs) have been proposed to infer directed graphs from time series data [32]. This method maximizes the Bayesian scoring function over alternative network models. A prior knowledge or assumption of the hierarchical structure is needed. Furthermore, it is computationally prohibitive to go through all the possible models as the cardinality of the model space grows exponentially with the number of genes. Therefore, the DBNs method is not capable of handling large networks. Linear dynamic system is also proposed [31] to model gene networks based on time series data. It is essentially a linear autoregressive model allowing extra hidden variables. It assumes the linear relationship between genes which may not be tenable in practice. In contrast, our method focuses on exploring pairwise dependencies between the genes. The computational complexity is much less demanding than the DBNs method. This enables us to analyze much larger datasets than the DBNs method. Compared to linear dynamic system method, our method can model nonlinear and combinatorial relationships among genes, which is more realistic than the linear assumptions. In conclusion, the computational simplicity in the algorithm, the capability of handling large dataset, modeling nonlinear relationships, and no prior assumptions of the network structure are the advantages of our method. Nevertheless, the limitation of our method is that it only produces undirected graph. In practice, our method can be used as the first screening method to identify the potential candidate edges. Once we narrow down our candidate genes list to a small set, we can use the DBNs method to study a finer structure of the network with additional details such as directions.
